# Long non-coding RNA BBOX1-antisense RNA 1 enhances cell proliferation and migration and suppresses apoptosis in oral squamous cell carcinoma via the miR-3940-3p/laminin subunit gamma 2 axis

**DOI:** 10.1080/21655979.2022.2059982

**Published:** 2022-05-04

**Authors:** Chunguang Zhao, Wei Shi, Min Chen

**Affiliations:** a Department of Stomatology, the Sixth Hospital of Wuhan, Affiliated Hospital of Jianghan University, Wuhan, Hubei, China; b Department of Otolaryngology, Tongji Hospital Affiliated to Tongji Medical College of Hust, Wuhan, Hubei, China

**Keywords:** BBOX1-AS1, miR-3940-3p, LAMC2, proliferation

## Abstract

Long non-coding RNAs (lncRNAs) play an essential role in oral squamous cell carcinoma (OSCC). We aimed to demonstrate the effects of lncRNA gamma-butyrobetaine hydroxylase 1 (BBOX1)-antisense RNA 1 (AS1) in OSCC and its regulatory mechanisms. The levels of BBOX1-AS1, microRNA (miR)-3940-3p, and laminin subunit gamma 2 (LAMC2) in OSCC were determined using reverse transcription-quantitative polymerase chain reaction. The correlations among BBOX1-AS1, miR-3940-3p, and LAMC2 were validated using luciferase, pull-down, and RNA immunoprecipitation assays. Cell proliferation, migration, and apoptosis were examined. BBOX1-AS1 and LAMC2 were notably overexpressed in OSCC, while miR-3940-3p showed the opposite trend. BBOX-1-AS1 silencing reduced the cell proliferation and migration, while promoting apoptosis. Mechanistically, BBOX1-AS1 modulates LAMC2 expression by competitively binding to miR-3940-3p. miR-3940-3p inhibition alleviated the inhibitory effects of BBOX1-AS1 deficiency on OSCC development. LAMC2 knockdown reversed these changes. Our results revealed that BBOX1-AS1 promotes the malignant phenotype of OSCC cells via the upregulation of LAMC2 expression by targeting miR-3940-3p, indicating that BBOX1-AS1 may be a novel target for OSCC intervention.

## Graphical abstract

**Figure:**
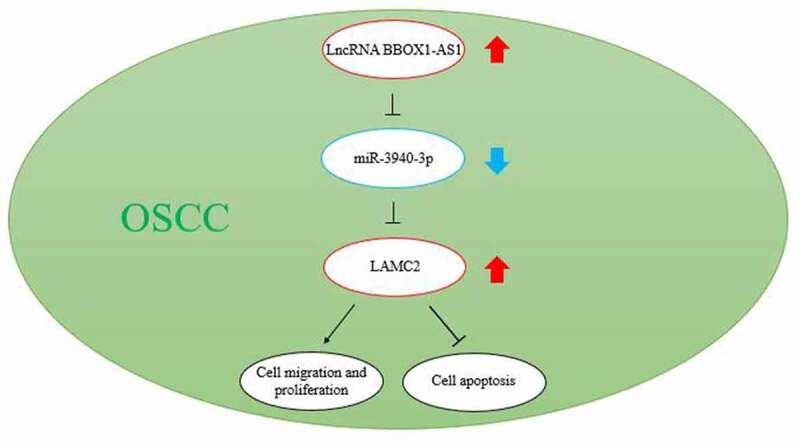

## Highlights

- BBOX1-AS1 and LAMC2 are overexpressed, while miR-3940-3p is downregulated in OSCC.
- BBOX1-AS1 promotes the malignancy of OSCC by targeting miR-3940-3p.
- BBOX1-AS1, miR-3940-3p, and LAMC2 form a ceRNA network to regulate OSCC progression.

## Introduction

Oral squamous cell carcinoma (OSCC) is a severe malignancy that mainly affects the head and neck [1]. According to the global cancer statistics of 2019, the 5-year survival rate for patients with OSCC was only approximately 50%. Targeting drugs show good efficacy against most tumors because of their high selectivity and low toxicity [2,3]. The use of targeted therapy has led to the development of novel approaches for the treatment of OSCC in patients with advanced cancer. Therefore, the identification of prognostic biomarkers and novel targets for targeted therapy is of great significance to understand the pathogenesis of OSCC.

Recently, a transcriptome investigation revealed that long non-coding RNAs (lncRNAs) are putative players in the tumorigenesis of most tumors [4]. LncRNA SNHG1 expression levels are elevated in OSCC cells and promote tumor cell growth by downregulating the expression of fucosyltransferase 8 by targeting microRNA (miR)-186 [5]. MYC-induced lncRNA enhances the invasion of OSCC cells by modulating the Wnt/β-catenin pathway [6]. LncRNA gamma-butyrobetaine hydroxylase 1 (BBOX1)-antisense RNA 1 (AS1) exists as an oncogene in most tumor cells. Abnormally high expression levels of BBOX1-AS1 are observed in most cancers, such as lung [7], cervical [8], and esophageal cancers [9], and BBOX1-AS1 is strongly linked to the overall survival of patients with these cancers, which suggests that targeting BBOX1-AS1 may be used as a therapeutic approach for cancer treatment. However, the regulatory effects of BBOX1-AS1 on OSCC development have not yet been elucidated.

miRNAs consist of 18–22 nt endogenous non-coding RNA molecules [10]. miRNAs play significant roles in various physiological and pathological processes by regulating the mRNA expression, and aberrant miRNA expression is closely related to the initiation and progression of cancer [11]. Fu *et al*. found that miR-155 acts as a tumor promoter in the proliferation of OSCC cells by regulating cyclin dependent kinase inhibitor 1B [12]. Moreover, Rastogi *et al*. demonstrated that the downregulation of miR-377 expression facilitates OSCC cell migration by targeting HDAC9 [13]. BBOX1-AS1 acts as an miR-3940-3p sponge that participates in the regulation of gastric cancer progression [14]. However, the relationship between miR-3940-3p and BBOX1 in OSCC has not yet been studied.

Laminin subunit gamma 2 (LAMC2), a laminin extracellular matrix protein, is a cross-shaped heterotrimer composed of one heavy (α) and two light (β and γ) chains. High expression of LAMC2 promotes tumor metastasis via the epithelial–mesenchymal transition and epidermal growth factor receptor pathways [15]. In addition, abnormal LAMC2 expression in various cancers, including colorectal cancer [16], bladder cancer [17], and lung adenocarcinoma [18], is closely correlated with poor prognosis. These findings indicate that LAMC2 may serve as a potential tumor marker. Only a few studies have been conducted on LAMC2 expression in OSCC; therefore, the upstream regulatory mechanisms of *LAMC2* as a target gene in OSCC need to be elucidated in future studies.

To study the functions of lncRNA BBOX1-AS1 and its underlying mechanisms in OSCC, we constructed an lncRNA-associated competing endogenous RNA (ceRNA) network in OSCC and verified that BBOX1-AS1 regulated OSCC development by sponging miR-3940-3p and regulating LAMC2 expression. Our results provide an effective diagnostic marker for OSCC.

## Materials and methods

### Clinical specimens

Fifty OSCC and paracancerous tissues were acquired from the patients hospitalized at the Department of Stomatology of the Sixth Hospital of Wuhan, Affiliated Hospital of Jianghan University (Wuhan, China). The tumor tissue was identified as OSCC and the tumor-adjacent tissue was identified as normal tissue by pathologists. Written informed consent was obtained from each patient before enrollment in this study. The use of clinical tissues was approved by the Hospital Research Ethics Committee.

### Cell culture

Normal human oral keratinocyte (NHOK) cells and four OSCC cell lines (SAS, OECM1, OC3, and HSC3) were purchased from BeNa Culture Collection (Beijing, China). All cells were maintained in RPMI-1640 medium containing 10% fetal bovine serum (ThermoFisher, USA) and incubated at 37°C with 5% CO_2_. After 2–3 stable generations, the cells were used for subsequent experiments.

### Cell transfection

The short hairpin RNAs targeting BBOX1-AS1 (sh-BBOX1-AS1) and LAMC2 (sh-LAMC2), matched scramble control (sh-NC), miR-3940-3p mimic/inhibitor, and mimic/inhibitor NC were assembled by Gene Copoeia (Guangzhou, China). Cell transfection was conducted using Lipo2000 (Invitrogen, USA), according to the manufacturer’s instructions. After 48 h, polymerase chain reaction (PCR) was performed to assess the transfection efficiency.

### Reverse transcription-quantitative PCR (RT-qPCR)

TRIzol reagent (Sigma-Aldrich, USA) was used to extract the total RNA from tissues and cells. Next, reverse transcription of RNA samples was performed to synthesize cDNA using the TaqMan MicroRNA Reverse Transcription Kit (Sigma-Aldrich, USA), according to the manufacturer’s instructions. For PCR amplification, a Light Cycler instrument (Bio-Rad, Hercules, USA) was used to prepare the reaction system, followed by detection using the SYBR Green PCR kit (Enzynomics, Korea) and SYBR Premix Ex Taq II kit (Takara, USA). Relative expression was calculated using the 2^−ΔΔ^Ct method, and normalized to glyceraldehyde‐3-phosphate dehydrogenase (GAPDH) and uracil 6 [19]. Primer sequences are listed in Table 1.

**Table 1.** The sequences of the primers in this study

Primer	Sequences
miR-361-3p	Forward: 5’-UCCCCCAGGUGUGAUUCUGAUUU-3’
	Reverse: 5’-GCAAATCAGAATCACACCTG-3’
miR-3940-3p	Forward: 5’-CTCAAGGACCACCGCATC-3’
	Reverse: 5’-ATCTGCAAGGGACAGCACAG-3’
BBOX1-AS1	Forward: 5’-TGTGTGTTTCCTGAGGCCTC-3’
	Reverse: 5’-CGCCTCTCTTGGAACACCTT-3’
LAMC2	Forward: 5’-GCCTTTTGGCACCTGTATTC-3’
	Reverse: 5’-CAGGATTCTCATCCCCTGAA-3’
Bax	Forward: 5’-GGTTGCCCTCTTCTACTTT-3’
	Reverse: 5’-AGCCACCCTGGTCTTG-3’
Bcl-2	Forward: 5’-ACTTTGCAGAGATGTCCAGT-3’
	Reverse: 5’-CGGTTCAGGTACTCAGCAT-3’
GAPDH	Forward: 5’-CCATGTTCGTCATGGGTGTG-3’
	Reverse: 5’-GGTGCTAAGCAGTTGGTGGTG-3’
U6	Forward: 5’-CGCTTCGGCAGCACATATACTA-3’
	Reverse: 5’-TATGGAACGCTTCACGAATTTGC-3’

### Cell proliferation assay

Cells (2 × 10^4^ cells/well) were maintained in a 96-well plate and cultured for different incubation periods (24, 48, and 72 h). Next, 10 μL of the cell counting kit (CCK)-8 solution (Abcam, USA) was added to the cells for 1 h [20]. Finally, the absorbance at 450 nm was measured using a microplate reader (Syngene, USA) to analyze the relative cell viability.

### BrdU proliferation assay

After transfection, the cells (1 × 10^4^ cells per well) were maintained in a 96-well plate overnight. The cells were then exposed to BrdU labeling reagent for 12 h, and it was followed by an assay using the ELISA BrdU kit (11,647,229,001; Roche, USA). The optical density of the cells at 450 nm was measured at different time-points using a microplate reader (Syngene, USA).

### Transwell assay

A transwell assay was performed to detect the cell migration, as described previously [21]. Transfected cells (2 × 10^4^ cells/mL) were grown in 100 μL of serum-free RPMI medium and added to a matrigel-coated transwell upper chamber (8 μm; BD Biosciences, USA). Then, 600 μL of complete RPMI medium was added to the lower chamber. Subsequently, 4% formaldehyde was used to fix the migrated cells and 0.5% crystal violet was used to stain the cells. Finally, the number of cells was counted using an inverted microscope (Nikon, Tokyo, Japan).

### Fluorescence in situ hybridization (FISH)

The LINC01207 FISH probe was designed and synthesized by RiboBio (Guangzhou, China). Briefly, the sections were deparaffinized, dehydrated in 100% ethanol, and dried. The slides were baked in a 60°C oven for 60 min and then dewaxed in xylene for 15 min. Then, 10 μL of hybridization buffer was added to the glass sample, and 1 μL probe was added to the hybridization buffer, placed in 46°C incubator, and hybridized for 1.5 h. After hybridization, the slides were washed with saline-sodium citrate buffer and mounted in an anti-fade solution containing DAPI. Images were obtained using a confocal fluorescence microscope (Leica, Germany) [22].

### Luciferase reporter assays

Luciferase reporter vectors of lncRNA BBOX1-AS1 and LAMC2 wild-type (WT) 3ʹ-untranslated regions (3'-UTR) and the corresponding mutant (Mut) 3'-UTR vectors containing the miR-3940-3p binding site were constructed. WT reporter genes (BBOX1-AS1-WT and LAMC2-WT) or the corresponding Mut type (BBOX1-AS1-Mut and LAMC2-Mut) genes were delivered into SAS and OC3 cells with miR-3940-3p mimic or NC. On the next day, the luciferase reporter system (Promega, USA) was used to detect the luciferase activity [23].

### RNA immunoprecipitation (RIP) assay

Using the RIP assay kit (Millipore, USA), as previously described [24], the cells from each group were lysed with RIP buffer. Antibodies against argonaute 2 (AGO2) and immunoglobulin G (IgG) were incubated with magnetic beads to prepare the antibody-coated beads. Supernatants of cell lysates were incubated with AGO2 or IgG antibody-coated magnetic beads for 4 h at 4°C. RNA complexes on the beads were isolated and used for RT-qPCR analysis.

### RNA Pull-down assay

RNA pull-down assay was performed as previously described [25]. SAS and OC3 cells were incubated with biotinylated-miR-3940-3p and biotinylated-NC for 48 h and lysed with RNase. M-280 streptavidin magnetic beads (Sigma, USA) were incubated with the cell lysate. The beads were washed twice with a low-salt wash buffer. RNA-binding proteins were purified, and their relative expression was used for RT-qPCR analysis.

### Western blotting

Western blotting was performed as per the previously published procedure [26]. Total protein from the cells was lysed using RIPA buffer (Santa Cruz Biotechnology, USA). Approximately 20 μg of protein extract was loaded onto 12% SDS-PAGE and transferred to PVDF membranes (Millipore, USA). The membranes were then exposed to 5% skim milk and probed with primary antibodies against LAMC2 (1:1000; ab274376; Abcam), B-cell lymphoma (Bcl)-2 (1:1000; ab32124), Bcl-2-associated X (Bax) (1:1000; ab32503), and GAPDH (1:1000; #5174; Cell Signaling Technology Inc., USA) at 4°C overnight, followed by examination with the corresponding horseradish-peroxidase-conjugated secondary antibodies (1:1000; SA205; Solarbio, China) at 37°C for 1 h. ECL kit (Najm Biotech ECL, Iran) was used to treat the membranes.

### Statistical analysis

IBM SPSS software (version 19.0) was used for statistical analysis, and GraphPad Prism (version 8) was used for mapping. The data obtained from at least three independent experiments are represented as the mean ± SD. The correlations among BBOX1-AS1, miR-3940-3p, and LAMC2 expression levels in OSCC tissues were assessed using Pearson’s analysis. Differences between groups were compared using the Student’s *t*-test or analysis of variance, in which a P-value < 0.05 was considered to be statistically significant.

## Results

In this study, we investigated the effects of lncRNA BBOX1-AS1 on OSCC and its regulatory mechanisms. Our data revealed significant upregulation of BBOX1-AS1 and LAMC2 expression levels, and downregulation of miR-3940-3p expression in OSCC. Moreover, we found that BBOX1-AS1 facilitated the proliferation and migration, while suppressing the apoptosis of OSCC cells by upregulating LAMC2 expression by targeting miR-3940-3p. Overall, our findings demonstrate the role of the BBOX1-AS1/miR-3940-3p/LAMC2 axis in OSCC and suggest that these molecules may be used as novel targets for OSCC therapy.

### LAMC2 is potentially regulated by BBOX1-AS1/miR-3940-3p and affects oral cancer progression

**Figure 1.**
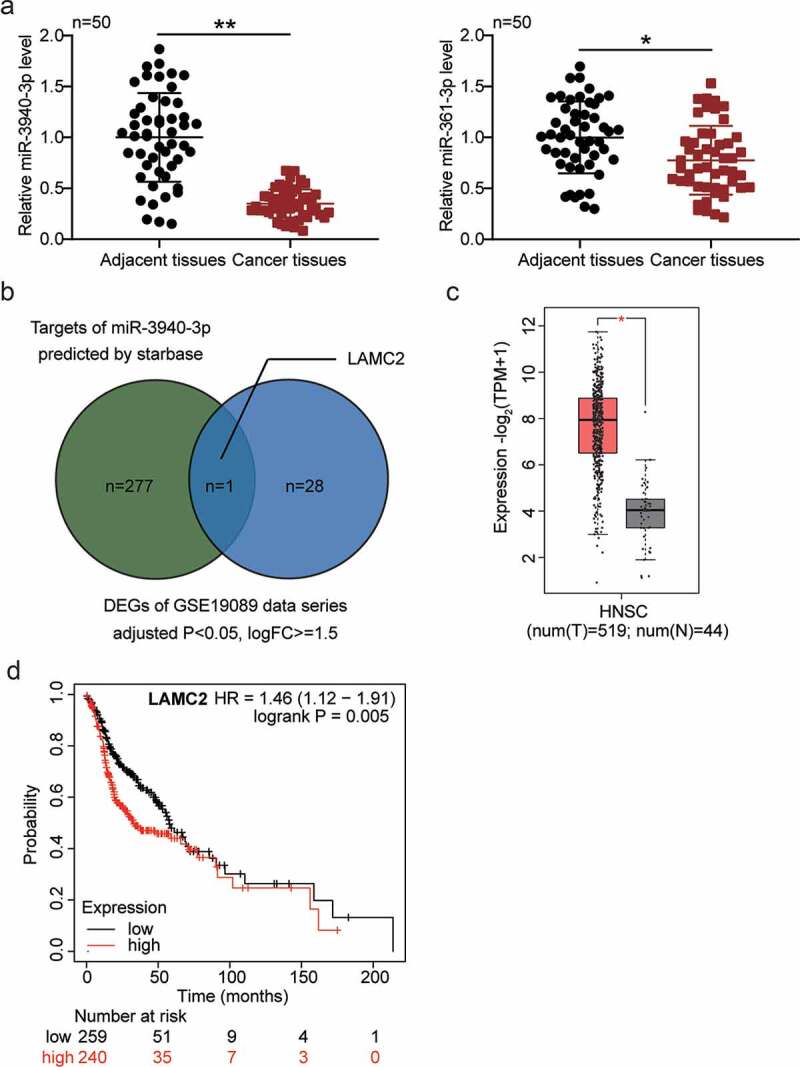
**LAMC2 was selected to be potentially modulated by BBOX1-AS1/miR-3940-3p thus affecting oral cancer progression**. (a) The expression of miR-3940-3p and miR-361-3p in our collected tissue samples. (b) The intersection between the predicted mRNA list of miR-3940-3p and the differentially expressed mRNAs in oral cancer (data obtained from GSE19089 data analysis with adjusted P < 0.05 and logFC≥1.5). (c) LAMC2 expression in head and neck squamous cell carcinoma (HNSCC) of GEPIA database. (d) Overall survival outcome of distinct LAMC2 expression levels in head and neck squamous cell carcinoma. *p < 0.05 and **p < 0.01.

BBOX1-AS1 has two downstream miRNA targets, miR-3940-3p and miR-361-3p. The latter has been reported to be a potential oncogene in oral cancer [27], whereas the former has not yet been studied in oral cancer. In addition, we detected significantly downregulated miR-3940-3p levels in oral cancer cells (Figure 1(a)). By intersecting the predicted mRNAs of miR-3940-3p using the starBase algorithm and the differentially expressed mRNAs in oral cancer by analyzing GSE19089 with adjusted P < 0.05 and logFC ≥ 1.5, LAMC2 was identified (Figure 1(b)). LAMC2 was found to be overexpressed in head and neck squamous cell carcinoma (Figure 1(c), data obtained from GEPIA database). The overall survival analysis showed that a higher level of LAMC2 predicted a poorer survival outcome (Figure 1d, data obtained from http://kmplot.com/analysis [28]). In addition, LAMC2 is known to enhance OSCC progression [27,29,30]. However, whether LAMC2 is involved in ceRNA and regulates oral cancer progression remain unclear.

### BBOX1-AS1 is upregulated in OSCC and its downregulation exerts oncosuppressive effects on OSCC cells

**Figure 2.**
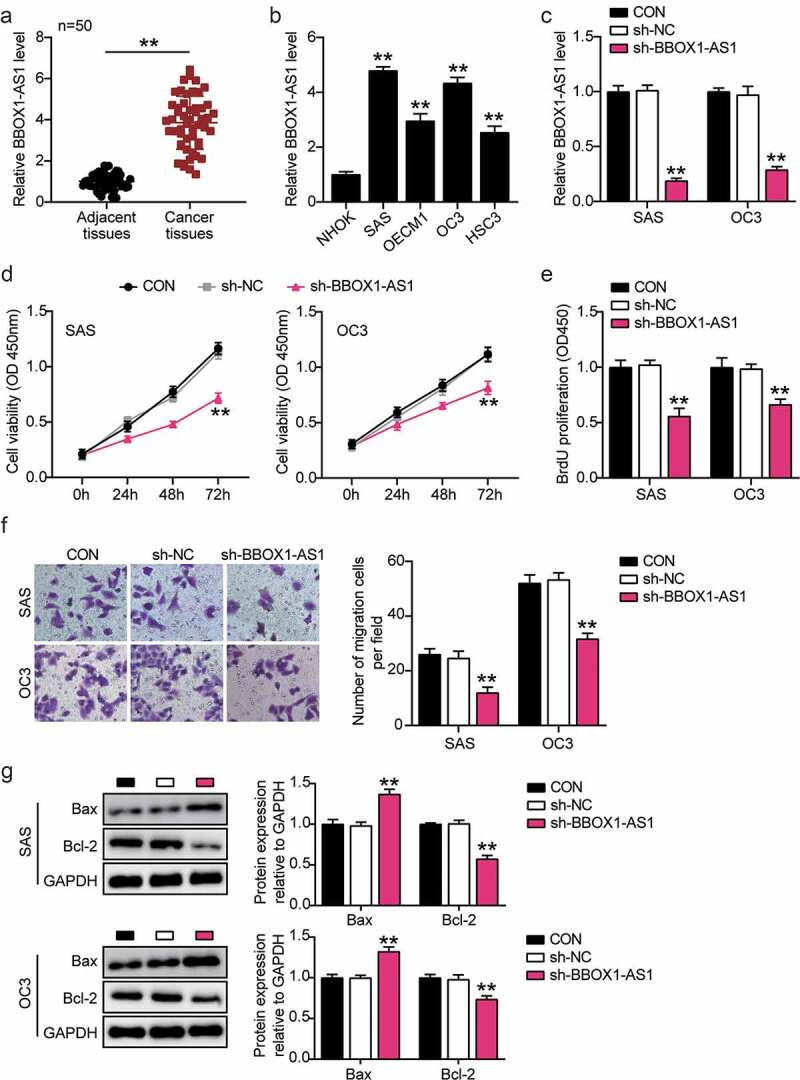
**BBOX1-AS1 was upregulated in OSCC and downregulation of BBOX1-AS1 exerted its oncosuppressive functions in OSCC cells**. (a) BBOX1-AS1 mRNA expression in OSCC and adjacent normal tissues. **p < 0.01. (b) Expression of BBOX1-AS1 in normal human oral keratinocytes (NHOK) and four OSCC cells (SAS, OECM1, OC3 and HSC3) were analyzed using RT-qPCR. **p < 0.01 vs. NHOK. (c) RT-qPCR was employed to confirm the shRNA-mediated BBOX1-AS1 knockdown efficiency. **p < 0.01 vs. sh-NC. (d) Effect of shRNA for BBOX1-AS1 on the activity of SAS and OC3 cells was detected by CCK-8 assay. **p < 0.01 vs. sh-NC. (e) Effect of shRNA for BBOX1-AS1 on the proliferation of SAS and OC3 cells was detected by BrdU proliferation assay. **p < 0.01 vs. sh-NC. (f) Effect of shRNA for BBOX1-AS1 on the migration of SAS and OC3 cells was detected by Transwell assay. **p < 0.01 vs. sh-NC. (g) Effect of shRNA for BBOX1-AS1 on the expression of Bcl-2 and Bax in SAS and OC3 cells was tested by western blotting. **p < 0.01 vs. sh-NC.

RT-qPCR analysis confirmed the amplification of BBOX1-AS1 in OSCC tissues (Figure 2a). Moreover, BBOX1-AS1 was overexpressed in four OSCC cell lines (SAS, OECM1, OC3, and HSC3), with the highest expression in SAS and OC3 cells (Figure 2b). Therefore, SAS and OC3 cells were screened for follow-up experiments. Next, shRNA transfection was performed to downregulate BBOX1-AS1 expression in OSCC cells. In sh-BBOX1-AS1 cells, BBOX1-AS1 expression was the lowest, indicating successful transfection (Figure 2(c)). Subsequently, CCK-8 analysis showed that the knockdown of BBOX1-AS1 reduced the cell viability (Figure 2(d)). Similarly, BrdU experiments demonstrated that BBOX1-AS1 knockdown inhibited the cell proliferation (Figure 2(e)). In the transwell assay, BBOX1-AS1 deficiency significantly reduced the migration of SAS and OC3 cells (figure 2(f)). Finally, Bax expression levels were elevated, while Bcl-2 levels were decreased in OSCC cells with BBOX1-AS1 knockdown (Figure 2(g)). These data demonstrate that BBOX1-AS1 downregulation inhibits OSCC development.

### miR-3940-3p is a target gene of BBOX1-AS1

**Figure 3.**
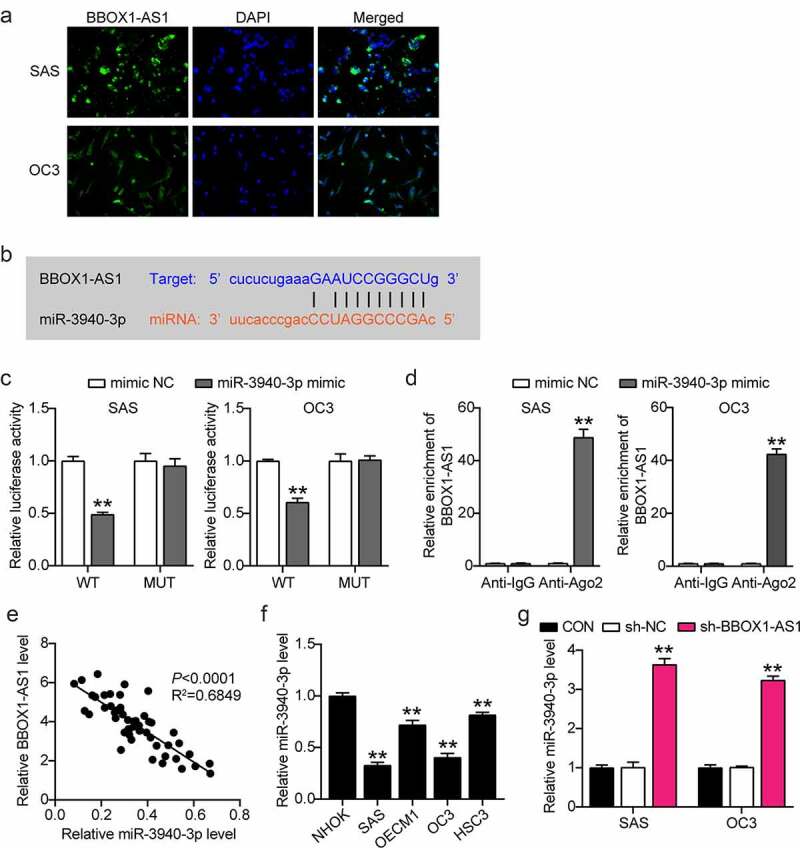
**MiR-3940-3p was a direct downstream target of BBOX1-AS1**. (a) FISH assay was performed to detect the subcellular location of BBOX1-AS1. (b) The binding sites between BBOX1-AS1 and miR-3940-3p. (c) The interaction between BBOX1-AS1 and miR-3940-3p was determined by a luciferase reporter assay. **p < 0.01 vs. mimic NC. (d) The targeting relationship between BBOX1-AS1 and miR-3940-3p was determined by the RIP assay. **p < 0.01 vs. Anti-IgG. (e) Correlation between BBOX1-AS1 expression and miR-3940-3p was analyzed by Spearman’s analysis. (f) Expression of miR-3940-3p in normal NHOK cells and four OSCC cells (SAS, OECM1, OC3 and HSC3) were analyzed by RT-qPCR. **p < 0.01 vs. NHOK. (g) Effect of shRNA for BBOX1-AS1 on the expression of miR-3940-3p was analyzed using RT-qPCR. **p < 0.01 vs. sh-NC.

To further study the regulatory role of BBOX1-AS1 in OSCC progression, we first detected the subcellular localization of BBOX1-AS1 using FISH assay, which showed that BBOX1-AS1 was mainly located in the cytoplasm of SAS and OC3 cells (Figure 3(a)), indicating that BBOX1-AS1 could act as a ceRNA via the spongy adsorption of miRNA. We subsequently analyzed the potential target genes of BBOX1-AS1 using starBase data. After a series of screenings, miR-3940-3p was selected as the target gene (Figure 3(b)). Next, luciferase assay results showed that the miR-3940-3p mimic considerably weakened the luciferase activity in OSCC cells transfected with the WT reporter vector of BBOX1-AS1, while luciferase activity was unaffected in cells transfected with the MUT reporter vector (Figure 3(c)). RIP results showed that large amounts of BBOX1-AS1 and miR-3940-3p were enriched by anti-AGO2 relative to anti-IgG antibodies (Figure 3(d)). These results demonstrate the relationship between BBOX1-AS1 and miR-3940-3p. Spearman’s correlation analysis showed that BBOX1-AS1 expression was negatively correlated with miR-3940-3p expression (Figure 3(e)). In addition, miR-3940-3p expression levels were significantly decreased in all four OSCC cell lines (figure 3(f)), and BBOX1-AS1 expression was negatively correlated with miR-3940-3p expression (Figure 3(g)). In general, miR-3940-3p was determined to be a target gene of BBOX1-AS1.

### BBOX1-AS1 exerts its oncogenic effects on OSCC cells by interacting with miR-3940-3p

**Figure 4.**
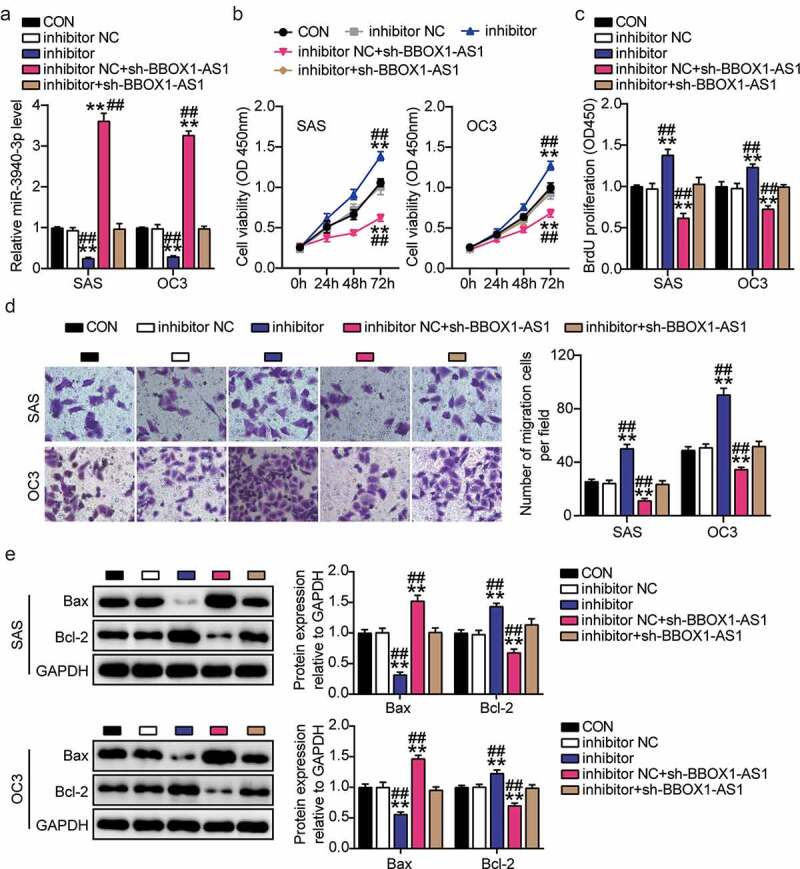
**BBOX1-AS1 exerted its oncogenic roles in OSCC cells by interacting with miR-3940-3p**. (a) MiR-3940-3p inhibitor transfection efficiency of SAS and OC3 cells was determined using RT-qPCR. (b) BBOX1-AS1 silencing results in the SAS and OC3 cell viability via CCK-8 assay. (c) Cell proliferation in SAS and OC3 cells after transfection with shRNA-BBOX1-AS1 miR-3940-3p inhibitor via BrdU proliferation assay. (d) Cell migration in SAS and OC3 cells co-transfected with shRNA-BBOX1-AS1 miR-3940-3p inhibitor was detected by Transwell assay. (e) Expression of Bcl-2 and Bax in SAS and OC3 cells co-transfected with shRNA-BBOX1-AS1 miR-3940-3p inhibitor was detected by western blotting. **p < 0.01 vs. inhibitor NC; ^##^p < 0.01 vs. inhibitor.

We verified the biological functions of the BBOX1-AS1/miR-3940-3p axis in OSCC cells. First, the miR-3940-3p inhibitor was used to knockdown miR-3940-3p in SAS and OC3 cells. The expression levels of miR-3940-3p were decreased in SAS and OC3 cells upon miR-3940-3p knockdown, whereas BBOX1-AS1 knockdown effectively alleviated these changes (Figure 4(a)). Subsequently, the cell proliferation activity assay revealed that the cell proliferation was remarkably suppressed by the downregulation of BBOX1-AS1 expression, but aggravated by miR-3940-3p inhibition (Figure 4(b–c)). We observed that cell migration was weakened in SAS and OC3 cells after transfection with BBOX1-AS1 knockdown cells, while the inhibitory effect of BBOX1-AS1 knockdown on migration was alleviated by an miR-3940-3p inhibitor (Figure 4(d)). Finally, BBOX1-AS1 knockdown reduced Bcl-2 expression and upregulated Bax expression, which were reversed by miR-3940-3p silencing (Figure 4(e)). These results indicate that BBOX1-AS1 promotes OSCC cell growth partly by inhibiting miR-3940-3p expression.

### miR-3940-3p is a negative upstream regulator of LAMC2

**Figure 5.**
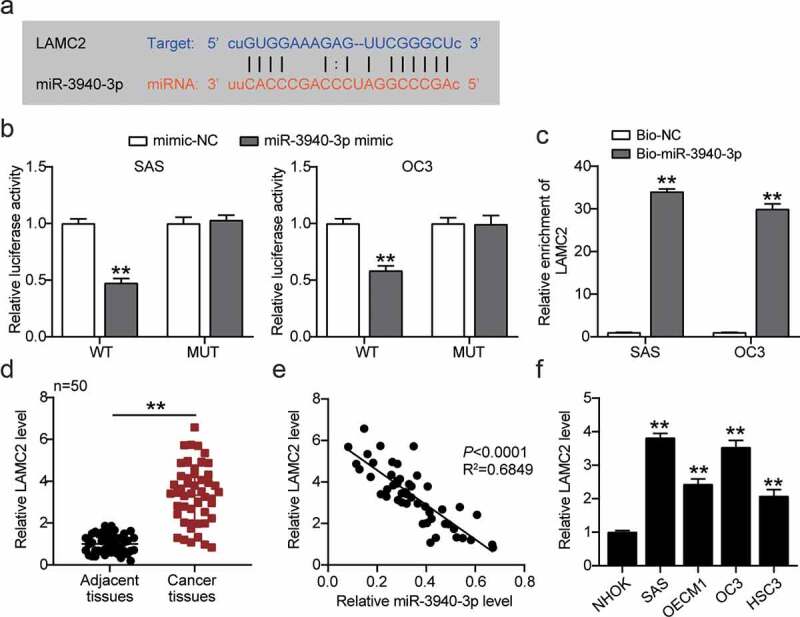
**MiR-3940-3p was a negative upstream regulator of LAMC2**. (a) The binding sites between miR-3940-3p and LAMC2. (b) The interaction of miR-3940-3p and LAMC2 was validated using a luciferase reporter assay. **p < 0.01 vs. mimic NC. (c) The targeting relationship between BBOX1-AS1 and miR-3940-3p was determined by a pull-down assay. **p < 0.01 vs. Bio-miR-3940-3p. (d) Expression of LAMC2 in OSCC tissues and normal tissues was detected by RT-qPCR. **p < 0.01. (e) Correlation between expression levels of miR-3940-3p and LAMC2 was analyzed by Spearman’s analysis. (f) Expression of LAMC2 in normal NHOK cells and four OSCC cells (SAS, OECM1, OC3 and HSC3) were analyzed using RT-qPCR. **p < 0.01 vs. NHOK.

To further illustrate its regulatory mechanisms, *LAMC2* was screened as an underlying downstream gene of miR-3940-3p using starBase (Figure 5(a)). Relative fluorescence activity was markedly reduced in LAMC2 WT SAS and OC3 cells transfected with miR-3940-3p (Figure 5(b)). RNA pull-down assay showed that miR-3940-3p could directly bind to LAMC2 (Figure 5(c)). In addition, LAMC2 expression was upregulated in OSCC, and miR-3940-3p expression was negatively correlated to that of LAMC2 (Figure 5(d–f)).

### Downregulation of LAMC2 counteracts the effects of miR-3940-3p knockdown on OSCC cells

**Figure 6.**
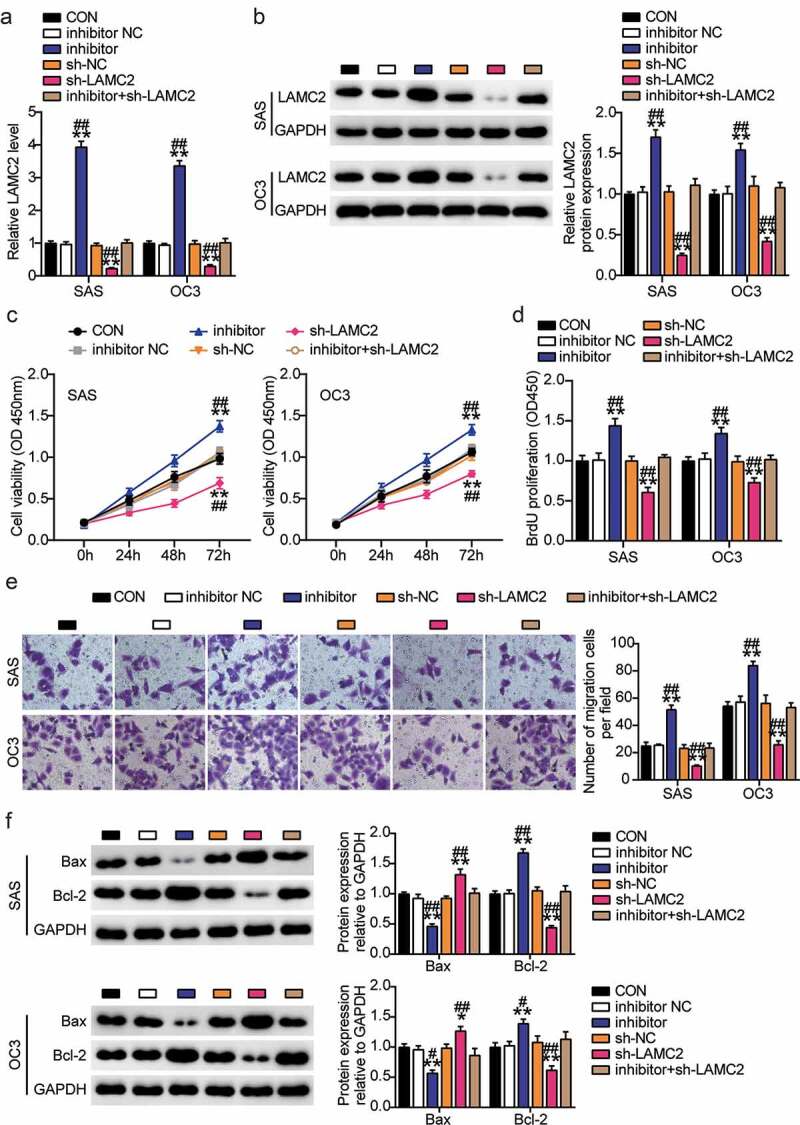
**Downregulation of LAMC2 counteracted the effect of miR-3940-3p knockdown on OSCC cells**(a) LAMC2 shRNA knockdown efficiency was detected by RT-qPCR. (b) LAMC2 shRNA knockdown efficiency was validated using a western blot assay. (c) MiR-3940-3p inhibitor and LAMC2 silencing results in the SAS and OC3 cell viability via CCK-8 assay. (d) Cell proliferation in SAS and OC3 cells after transfection with miR-3940-3p inhibitor and shRNA-LAMC2 via BrdU proliferation assay. (e) Cell migration in SAS and OC3 cells co-transfected with miR-3940-3p inhibitor and shRNA-LAMC2 was detected by Transwell assay. (f) Bcl-2 and Bax protein expressions in transfected SAS and OC3 cells were assessed using western blotting. **p < 0.01 vs. inhibitor NC; ^##^p < 0.01 vs. sh-NC.

Expression analysis showed that miR-3940-3p inhibitor enhanced LAMC2 expression, but this effect could be reversed by LAMC2 knockdown (Figure 6(a, b)). CCK-8 and BrdU experiments showed that downregulation of miR-3940-3p expression facilitated OSCC cell proliferation, which was abolished by LAMC2 silencing (Figure 6(c, d)). Transwell assay showed that LAMC2 knockdown could counteract the promoting effect of miR-3940-3p inhibitor on the migration of OSCC cells (Figure 6(e)). Additionally, Bcl-2 expression levels were increased, while those of Bax were decreased in the SAS and OC3 cells transfected with the miR-3940-3p inhibitor. LAMC2 knockdown exerted the opposite effect (figure 6(f)). These results indicated that LAMC2 is a target of miR-3940-3p.

### BBOX1-AS1 enhances the proliferation and migration, and suppresses the apoptosis of OSCC cells by regulating LAMC2 expression

**Figure 7.**
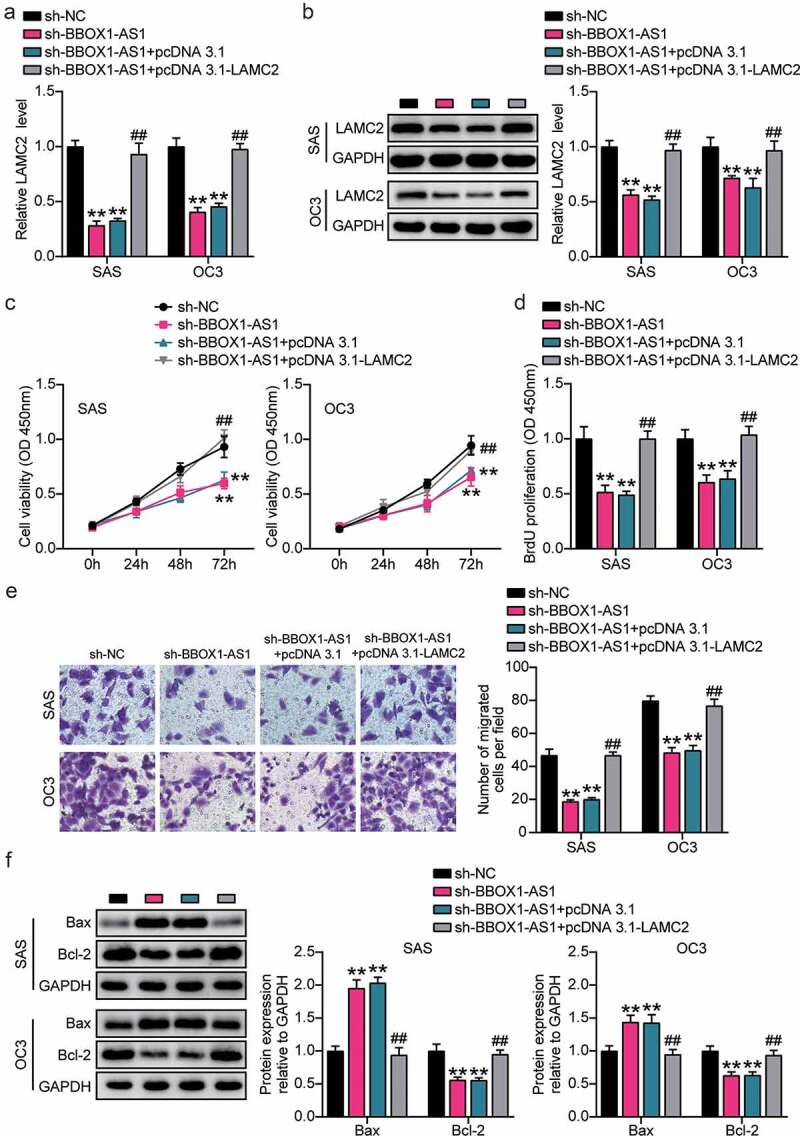
**BBOX1-AS1 facilitated the proliferation, migration and suppressed apoptosis of OSCC cells through regulating LAMC2**. (a) RT-qPCR was used to detect the LAMC2 level in SAS and OC3 cells after transfecting sh-BBOX1-AS1 and pcDNA3.1- LAMC2. (b) Western blot assay was used to detect the LAMC2 level in SAS and OC3 cells after transfecting sh-BBOX1-AS1 and pcDNA3.1- LAMC2. (c) Cell viability was detected by CCK-8 assay after after transfecting sh-BBOX1-AS1 and pcDNA3.1- LAMC2. (d) Cell proliferation was detected in SAS and OC3 cells after transfecting sh-BBOX1-AS1 and pcDNA3.1- LAMC2 via BrdU proliferation assay. (e) Cell migration was detected by Transwell assay after transfecting sh-BBOX1-AS1 and pcDNA3.1- LAMC2. (f) Bcl-2 and Bax protein expressions in transfected SAS and OC3 cells was were assessed using western blotting. **p < 0.01 vs. sh-NC; ##p < 0.01 vs. sh- BBOX1-AS1.

RT-qPCR and western blotting analysis revealed that the expression levels of LAMC2 were decreased in BBOX1-AS1 knockdown SAS and OC3 cells (Figure 7(a, b)). Subsequently, functional analysis showed that LAMC2 overexpression reversed the decrease in cell viability and proliferation induced by the downregulation of BBOX1-AS1 expression (Figure 7(c, d)). Transwell assay showed that the inhibitory effect of BBOX1-AS1 knockdown on cell migration could be alleviated by LAMC2 overexpression (Figure 7(e)). Additionally, LAMC2 overexpression counteracted the BE downregulation of Bcl-2 expression and upregulation of Bax expression induced by BBOX1-AS1 knockdown (figure 7(f)). Overall, BBOX1-AS1 may promote the expression of LAMC2 by targeting miR-3940-3p, thereby promoting OSCC development.

## Discussion

OSCC poses a serious threat to the overall survival of patients, and many patients are initially diagnosed with advanced OSCC [31]. Therefore, the search for novel key genes or biomarkers is necessary to facilitate the early diagnosis and better prognosis of patients with OSCC [32]. This study demonstrated that BBOX1-AS1 expression was elevated in OSCC. Functional *in vitro* experiments revealed that BBOX1-AS1 knockdown impaired OSCC cell growth, while promoting cell apoptosis. In addition, a novel mechanism for BBOX1-AS1 function is that it modulates miR-3940-3p/LAMC2 signaling to exert tumor-promoting effects.

As a newly discovered lncRNA, lncRNA BBOX1-AS1 has shown tumor-promoting effects in a wide range of tumors [33]. For example, BBOX1-AS1 expression is elevated in cervical cancer, and the growth and migration of cervical cancer cells can be repressed by BBOX1-AS1 silencing [34]. In colorectal cancer, BBOX1-AS1 acts as an oncogene and effectively accelerates cell proliferation and metastasis [35]. In ovarian cancer, BBOX1-AS1 knockdown suppresses tumor progression by decreasing the viability of cells [36]. These results suggest that BBOX1-AS1 may play a tumor-promoting role in major tumors and accelerate tumor progression. Therefore, we speculated that BBOX1-AS1 may also function as an antitumor gene in oral cancer. Our study showed that BBOX1-AS1 was highly expressed in OSCC. In *vitro* experiments showed that cell proliferation and migration were abolished, while apoptosis was promoted by BBOX1-AS1 silencing, indicating that BBOX1-AS1 may act as a pro-oncogenic factor to promote the progression of oral cancer.

Furthermore, lncRNAs not only directly participate in the regulation of gene expression, but also act as ceRNAs to interact with miRNAs, thereby regulating the levels of protein-coding target genes and affecting various physiological and pathological processes [37,38]. Lina *et al*. found that lncRNA CASC2 represses OSCC cell growth by sponging miR-21 [39]. Furthermore, lncRNA H1 enhances OSCC progression by silencing miR-138 [40]. miR-3940-3p introduced in our study belongs to a class of RNA molecules that have not yet been extensively studied. miR-3940-3p expression is higher in recurrent ovarian cancer than in primary ovarian cancer, and may be used as a biomarker for predicting recurrent ovarian cancer [41]. Interestingly, only one study exploring the specific biological function of miR-3940-3p indicated that miR-3940-3p expression levels were reduced in gastric cancer cells and that BBOX1-AS1 promoted gastric cancer cell progression by targeting miR-3940-3p [14]. Our data showed that miR-3940-3p expression levels are downregulated in OSCC cells. Luciferase and RIP assays confirmed that miR-3940-3p is the target gene of BBOX1-AS1, which is consistent with the above results. Moreover, BBOX1-AS1 expression was negatively correlated with miR-3940-3p expression. In rescue assays, miR-3940-3p knockdown significantly enhanced OSCC cell proliferation and migration, while promoting apoptosis. These changes were reversed by BBOX1-AS1 deficiency. Overall, BBOX1-AS1 promotes OSCC development by suppressing miR-3940-3p expression.

We further studied the downstream regulation pathway of BBOX1-AS1 in OSCC and constructed a complete lncRNA-miRNA-mRNA network. We analyzed the downstream target genes of miR-3940-3p in OSCC cells. LAMC2 is closely associated with various biological processes, including cell adhesion and tumor cell migration [42]. LAMC2 has been widely studied in oral cancer. Zhou *et al*. reported that LAMC2 is a crucial factor related to OSCC and that its silencing inhibits tumor growth in *vivo* [27]. Rong *et al*. demonstrated that miR-5580-3p inhibits OSCC tumorigenesis by downregulating the expression of LAMC2 [43]. Moreover, upregulation of LAMC2 is closely associated with the invasive phenotype of OSCC, suggesting that LAMC2 overexpression may aggravate the severity of OSCC [44]. In OSCC, LAMC2 functions as an oncogenic factor and promotes tumor cell growth. Ning *et al*. revealed that the lncRNA CASC9 promotes the malignancy of OSCC cells by sponging miR-545-3p to enhance LAMC2 expression [45]. However, the role of LAMC2 in the ceRNA network in the pathogenesis of OSCC remains to be elucidated. In our study, *LAMC2* was predicted to be the target gene of miR-3940-3p using starBase data. Results of GEPIA database analysis revealed that LAMC2 expression was increased in OSCC, and upregulation of LAMC2 expression was highly related to unfavorable patient outcomes. Subsequently, LAMC2 was confirmed to be directly targeted by miR-3940-3p, and the expression levels of LAMC2 and miR-3940-3p were negatively correlated with each other. Next, LAMC2 knockdown partially alleviated the promoting effect of the miR-3940-3p inhibitor on BBOX1-AS1-silenced OSCC cells. These data suggested that BBOX1-AS1 influences OSCC progression by modulating the miR-3940-3p/LAMC2 pathway.

This study has some limitations. First, more cellular experiments, including cell migration and invasion tests are needed to validate the effects of BBOX1-AS1 in OSCC by regulating the miR-3940-3p/LAMC2 axis. Second, in *vivo* experiments, such as xenograft tumor assays, must be carried out in follow-up studies. Finally, the correlation between BBOX1-AS1 and the clinicopathological characteristics of patients with OSCC requires further investigation in future studies.

## Conclusion

In conclusion, our results showed that BBOX1-AS1 facilitates oral cancer cell proliferation and migration and suppresses apoptosis by upregulating LAMC2 expression by targeting miR-3940-3p. Based on these findings, BBOX1-AS1 may be used as a potential target for OSCC treatment.
